# Pedestrian detection model based on Tiny-Yolov3 architecture for wearable devices to visually impaired assistance

**DOI:** 10.3389/frobt.2023.1052509

**Published:** 2023-03-16

**Authors:** Sergio-Uriel Maya-Martínez, Amadeo-José Argüelles-Cruz, Zobeida-Jezabel Guzmán-Zavaleta, Miguel-de-Jesús Ramírez-Cadena

**Affiliations:** ^1^ Centro de Investigación en Computación, Instituto Politécnico Nacional, Mexico City, Mexico; ^2^ Universidad de las Américas Puebla, Puebla, Mexico; ^3^ School of Engineering and Science, Tecnológico de Monterrey, Mexico City, Mexico

**Keywords:** Tiny YOLOv3, deep learning, visual impaired, image processing, graphic processing unit, pedestrian detection

## Abstract

**Introduction:**

Wearable assistive devices for the visually impaired whose technology is based on video camera devices represent a challenge in rapid evolution, where one of the main problems is to find computer vision algorithms that can be implemented in low-cost embedded devices.

**Objectives and Methods:**

This work presents a Tiny You Only Look Once architecture for pedestrian detection, which can be implemented in low-cost wearable devices as an alternative for the development of assistive technologies for the visually impaired.

**Results:**

The recall results of the proposed refined model represent an improvement of 71% working with four anchor boxes and 66% with six anchor boxes compared to the original model. The accuracy achieved on the same data set shows an increase of 14% and 25%, respectively. The F1 calculation shows a refinement of 57% and 55%. The average accuracy of the models achieved an improvement of 87% and 99%. The number of correctly detected objects was 3098 and 2892 for four and six anchor boxes, respectively, whose performance is better by 77% and 65% compared to the original, which correctly detected 1743 objects.

**Discussion:**

Finally, the model was optimized for the Jetson Nano embedded system, a case study for low-power embedded devices, and in a desktop computer. In both cases, the graphics processing unit (GPU) and central processing unit were tested, and a documented comparison of solutions aimed at serving visually impaired people was performed.

**Conclusion:**

We performed the desktop tests with a RTX 2070S graphics card, and the image processing took about 2.8 ms. The Jetson Nano board could process an image in about 110 ms, offering the opportunity to generate alert notification procedures in support of visually impaired mobility.

## 1 Introduction

According to the World Health Organization (WHO), More than two hundred million people worldwide have vision impairment problems WHO Noncommunicable Diseases Team (2019), like cataract, trachoma, refractive error, and other affections. Although these health problems are irreversible in some cases, in some others, visual impairment can be improved by treatment or rehabilitation. Projections show that global demand for eye care is set to surge in the coming years due to population growth, ageing, and lifestyle changes. For people in which the visual impairment is irreversible or requires rehabilitation, there are some assistive devices and health technologies that seek to improve their degree of independence, and well-being WHO Noncommunicable Diseases Team (2015).

In the arena of requirements fulfillment for assistive solutions for the visually impaired/blind, the work of Ntakolia et al. (2022) provides other example of assisted navigation for visually impaired individuals, focusing on outdoor cultural environments. Recent work made by Valipoor and de Antonio (2022) provide a rich survey highligthing the challenges in the field and systematic mapping of different approaches of computer vision-based assistive solutions. We recommend to take a look at this reference.

Tapu et al. (2018) state that the wearable assistive devices for the visually impaired can be divided into two main groups. The first one involves sensorial networks with electronic travel aids (ETAs), including technology-based infrared sensors, ultrasound sensors, global positioning system, radio frequency identification, and low-energy Bluetooth. The second group is composed of video camera-based ETAs, which are monocular video-based systems, stereo camera-based systems, and RGB-D camera-based systems.

Video camera-based ETAs result from the rapid evolution of low-cost wearable cameras, computer vision/machine learning algorithms, and embedded devices. In the work proposed by Cheng et al. (2018), the authors introduce a pedestrian crossing lights detection algorithm implemented in a portable computer with a colour camera as an aid for the visually impaired. Lin et al. (2018) propose a visual localizer for assisted navigation of the visually impaired; the system consists of a Realsense Camera, a GNSS processor, and a pair of earphones. Afif et al. (2020) developed an indoor detector for the visually impaired. The system is based on a deep convolutional neural network. In all these cases, the algorithms in charge of assisting the visually impaired are based on object detection (OD).

During the last decade, systems that perform OD have improved because it is one of the main computer vision tasks. OD it serves as the basis of many of its applications, such as semantic segmentation Xie et al. (2017), instance segmentation Cai and Vasconcelos (2019), pose estimation Ge et al. (2019), and object tracking Mahalingam and Subramoniam (2018). OD used specifically for pedestrian detection is the base of different tasks like person identification Li et al. (2018), person re-identification Wu et al. (2019), action recognition Mabrouk and Zagrouba (2018), and behavior analysis Yi et al. (2016), among others. Consequently, many applications that impact our daily lives can use pedestrian detection systems from intelligent surveillance systems to autonomous vehicles Arnold et al. (2019), and lately in medical devices Sathyamoorthy et al. (2020).

Pedestrian detection systems have the desirable characteristics of robustness while efficient in processing a large amount of data. Deep learning computational models have been used to achieve these goals. A deep learning model extracts a hierarchical representation from large-scale data Wang and Sng (2015). Convolutional Neural Networks (CNNs) are among the most popular deep learning architectures due to their ability to exploit spatial or temporal correlation in data Liu et al. (2019). Therefore, CNNs are ideal for image processing due to their high performance response in image segmentation, detection, retrieval-related tasks, and classification. A CNN adds convolutional layers to fully connected networks, typically consisting of convolutional operations, a batch normalization layer, a pooling layer and an activation function. The parameters and hyperparameters setup of a CNN model is essential to fit the application scenario.

There are two approaches in the object detection task using CNNs: one-stage methods and two-stage methods. In the latter, the models propose a set of regions of interest through a selective search. Then, a CNN classifier processes only the candidate regions. These include the family of Region-based Convolutional Neural Networks (R-CNN) Girshick et al. (2014). R-CNN was improved by unifying the input image with the Region of Interest (RoI), used as a Deep CNN input. The improved visual geometry group (VGG-16) network outputs the softmax probabilities, and the bounding box coordinates per RoI. Girshick (2015). Then, a Faster R-CNN was proposed by Liu et al. (2016). Even with these improvements, the inference time is around 0.32 s running on a desktop computer with a dedicated GPU.

In counterpart, one-stage methods are characterized by skipping the part of the proposed region, and instead, they directly provide the detected bounding box and the class to which it belongs. In general, these methods are more straightforward and faster but less robust than two-stage methods. The most representatives architectures of this type of models are the YOLO which is considered to be the first attempt to build a real-time object detection system Redmon et al. (2016), the Single Shot Detector (SSD) model Liu et al. (2016) which uses a pre-trained VGG16 as a feature extractor and uses the idea of pyramidal features for the efficient detection of objects at different scales. YOLOv2 is a Fully Convolutional Neural Network (FCNN), an architecture that can process images from different sizes and includes a batch normalization layer Ioffe and Szegedy (2015) on all the convolutional layers.

One of the significant disadvantages of the models presented is the need for a GPU to process images in ms, It was therefore necessary to apply new models or architectures or modify existing ones to mitigate this problem. That is why MobileNet was proposed by Howard et al. (2017). It is designed for mobile and embedded applications; Google released an improvement in 2018 called Mobilenet V2 Sandler et al. (2018). Tiny YOLO is a variant of the previously mentioned YOLO, and the release of an improvement over YOLOv3 was derived in Tiny YOLOv3, which is a simplified version architecture. YOLO-LITE Huang et al. (2018) is a real-time object detection model designed to run on portable devices with no GPU/TPU processors, based on the YOLOv2 model.

In summary, state-of-the-art CNN-based architectures such as R-CNN, VGG, Mobilenet, and YOLO series combined with SSD benefit OD and segmentation systems. Non-etheless, not every proposed model achieves the specific requirements for wearable devices that are robust and low-energy consumption. Therefore, this work considers the assessment of the well-known Tiny YOLOv3 architecture fitted for pedestrian detection. This assessment’s contribution has essential relevance for incorporating that model into embedded devices such as wearables for the visually impaired.

The rest of this document is organized as follows: Section 2, presents the Tiny YOLOv3 architecture that was employed in this work. Section 3 presents the mathematical background of the technique that enables the fitting of the Tiny YOLO architecture. Then, Section 4 outlines different embedded hardware platforms available to implement deep learning for pedestrian detection. Section 5, shows the experimental results achieved in this work. At last, Section 6 discusses the obtained results, concluding with Section 7.

## 2 The Tiny YOLOV3 architecture

**FIGURE 1:**
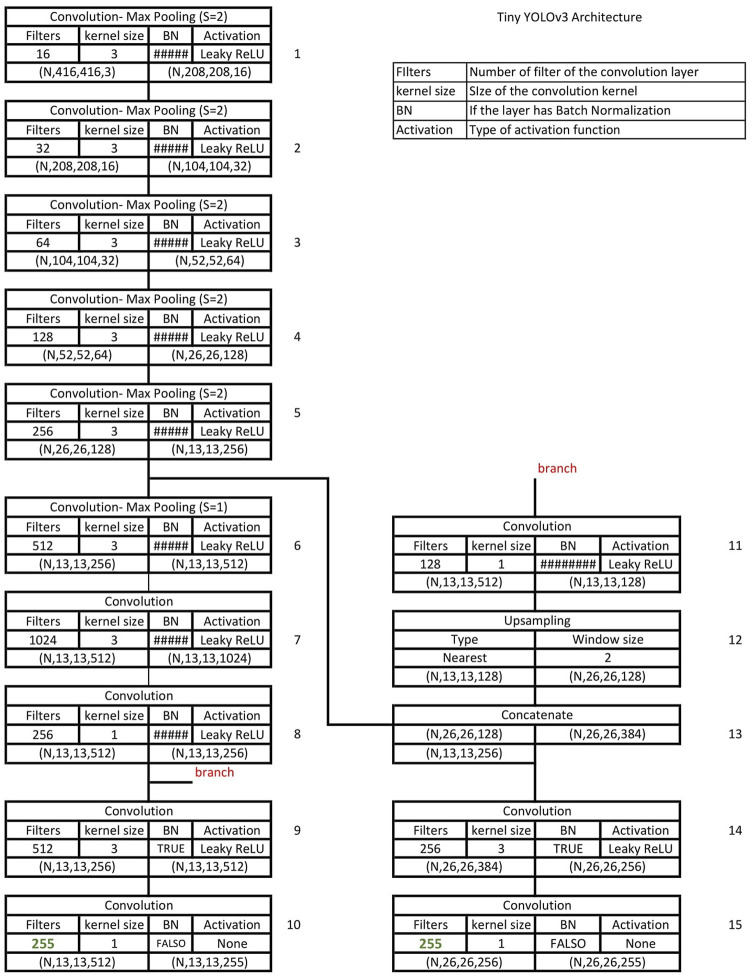
Tiny YOLOv3 architecture.

The Tiny YOLOv3 architecture, proposed by Redmon and Farhadi (2018) is designed for low-power devices based on novel ideas from object detection models as YOLOv2, YOLOv3, and FPN. The model was trained using MS COCO Dataset and VOC Dataset. It has a little over eight million trainable parameters and uses the same idea of YOLOv3 anchor boxes. The model uses six anchor boxes calculated with a k-means algorithm on the bounding boxes of the dataset. The complete architecture is shown in Figure 1.

The network comprises two essential parts, which are named feature extraction and detection. Feature extraction is performed by a series of convolutional layers that progressively increase the number of filters to extract more complex features. Each layer in this task uses Batch Normalization before the activation function Leaky ReLU, and uses max pooling for dimensionality reduction purposes. The network uses pyramidal feature extraction (block 13) concatenating the output of a convolution layer (block 8) with another (block 5) closer to the input layer as seen in Figure 1.

## 3 Adaptation of Tiny YOLOv3 using transfer learning

The transfer learning technique allows a trained model to be adapted for its use in a new scenario. In this case, the Tiny YOLOv3 is set up to detect 80 different class objects and trained initially with the COCO dataset. It is necessary to adjust some parameters to fit the Tiny YOLOv3 to detect only pedestrians more accurately. The fine-tuning process in the transfer learning technique involves retraining the model with a specific dataset and adjusting the loss function and the anchor boxes for pedestrian detection.

The dataset for the fine-tuning task collects people-related images from the COCO dataset and the Pascal VOC dataset. In this configuration, the collected dataset contains annotations with normalized pedestrian bounding boxes. A new set of anchor boxes for the only pedestrian class has to be determined; the following subsection presents its calculation. Finally, for the fitting process, a data augmentation methodology is employed to increase the accuracy of the model on different datasets (see Section 3.3 for details).

### 3.1 The training loss function

The loss function of the fitting process is an adaptation of the original model, omitting the minimization error for different objects. The loss function, presented in Eq. 1, optimizes the dimensions of the bounding box over each cell weighted by *λ*_*coord*_ when the function $1_{ij}^{\mathrm{obj}}$ aligns a cell *i* with an anchor box *j*, plus the balanced prediction of the object in the cell *i*. The balanced prediction is weighted by *λ*_*obj*_ and *λ*_*noobj*_ decreasing the effect of the unbalanced number of objects and no objects (background) in the dataset. The *o*_*i*_ takes the value of one when an object is in the cell *i*.

$$\begin{array}{ll} & L\left(t_{x},t_{y},t_{w},t_{h},o,\hat{t_{x}},\hat{t_{y}},\hat{t_{w}},\hat{t_{h}},\hat{o}\right)\\ & \;=\lambda_{\mathrm{coord}}\overset{S^{2}}{\underset{i=0}{\sum }}\overset{B}{\underset{j=0}{\sum }}1_{ij}^{\mathrm{obj}}\left[{\left(t_{\mathrm{xij}}-\hat{t_{\mathrm{xij}}}\right)}^{2}+{\left(t_{\mathrm{yij}}-\hat{t_{\mathrm{yij}}}\right)}^{2}\right]\\ & \;\;+\lambda_{\mathrm{coord}}\overset{S^{2}}{\underset{i=0}{\sum }}\overset{B}{\underset{j=0}{\sum }}1_{ij}^{\mathrm{obj}}\left[{\left(t_{\mathrm{wij}}-\hat{t_{\mathrm{wij}}}\right)}^{2}+{\left(t_{\mathrm{hij}}-\hat{t_{\mathrm{hij}}}\right)}^{2}\right]\\ & \;\;+\overset{S^{2}}{\underset{i=0}{\sum }}\overset{B}{\underset{j=0}{\sum }}\left[\lambda_{\mathrm{obj}}o_{i}*\log\left({\hat{o}}_{i}\right)+\lambda_{\mathrm{noobj}}\left(1-o_{i}\right)*\log\left(1-{\hat{o}}_{i}\right)\right] \end{array}$$ (1)

### 3.2 The anchor boxes for pedestrian detection

**FIGURE 2:**
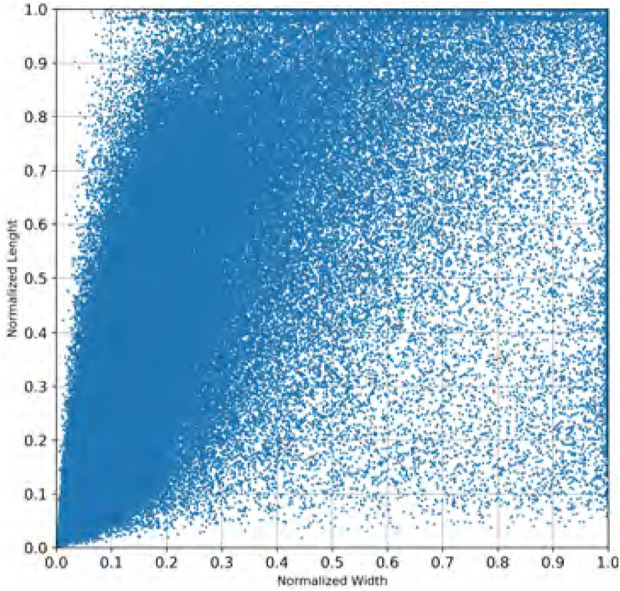
Bounding boxes in the training set.

The fine-tuning process requires a new set of anchor boxes fitted to pedestrian detection. To this aim, the filtered training dataset with people-related clips gave the bounding boxes annotations with pedestrians. Figure 2 shown all the boxes in the dataset that localize pedestrians. Each point in the figure represents a bounding box, and the coordinates represent the normalized width and the height of each box.

Following the Tiny YOLO v3 methodology for fitting, K-means clustering helped find the new normalized centroids of the anchor boxes. The purpose of this process is to maximize the intersection over union (IoU) measurement between the bounding boxes in the dataset and the anchor boxes according to Eq. 2. The number of clusters (k) and other initial parameters were empirically selected by experimenting five thousand times with different initial conditions.

$$k-means_{d}=1-IoU\left(bounding\;box,anchor\;box\right)$$ (2)

**FIGURE 3:**
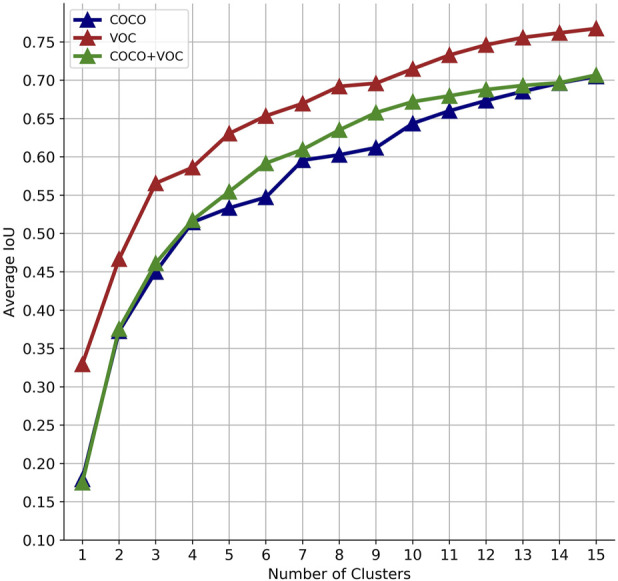
IoU for different number of clusters.

**FIGURE 4:**
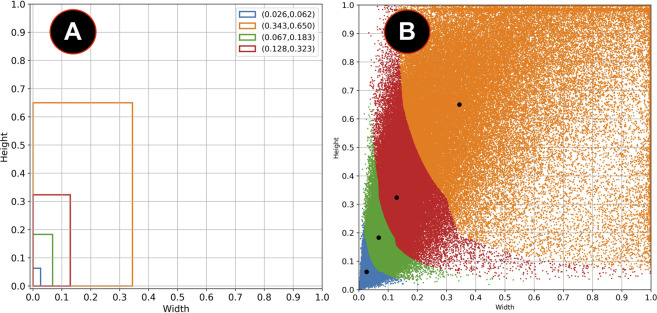
**(A)** Anchor boxes obtained with *k* = 4. **(B)** Clustered dataset with these anchor boxes.

**FIGURE 5:**
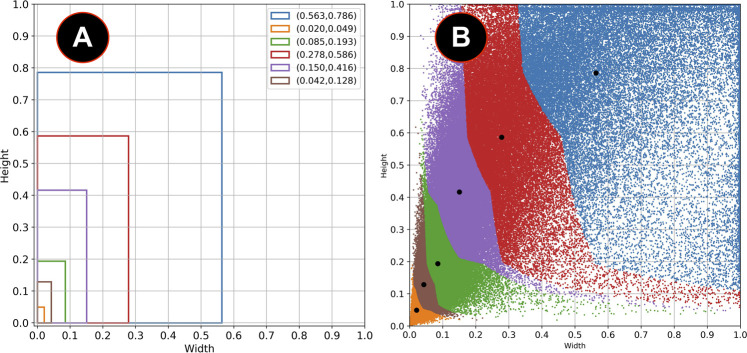
**(A)** Anchor boxes obtained with *k* = 6. **(B)** Clustered dataset with these anchor boxes.

Figure 3 shows the different values of IoU with the selected bounding boxes from the COCO and the VOC datasets and the combination of both COCO + VOC bounding boxes. Although the IoU increases significantly with *k* > 4, a good balance between the value of *k* and the number of parameters in the final layers of the model is to use *k* = 6. Figure 4A shows the clustered dataset with *k* = 4 centroids while Figure 4B shows the four anchor boxes found. Figure 5A shows the dataset clustered with *k* = 6 centroids whilst Figure 5B shows the six anchor boxes found.

### 3.3 Dataset augmentation

One of the most common techniques to avoid overfitting in deep neural networks is data augmentation. Data augmentation consists of applying different transformations to the images of the original dataset creating a new training dataset. The image transformations available are geometric (rotations, axis translations, horizontal or vertical flips, among others) and pixel-level (addition of noise, color transformations and others). For object detection, changes must be applied to the bounding boxes when necessary; for example, the rotation of the bounding box is similar to the rotation of the image.

**FIGURE 6:**
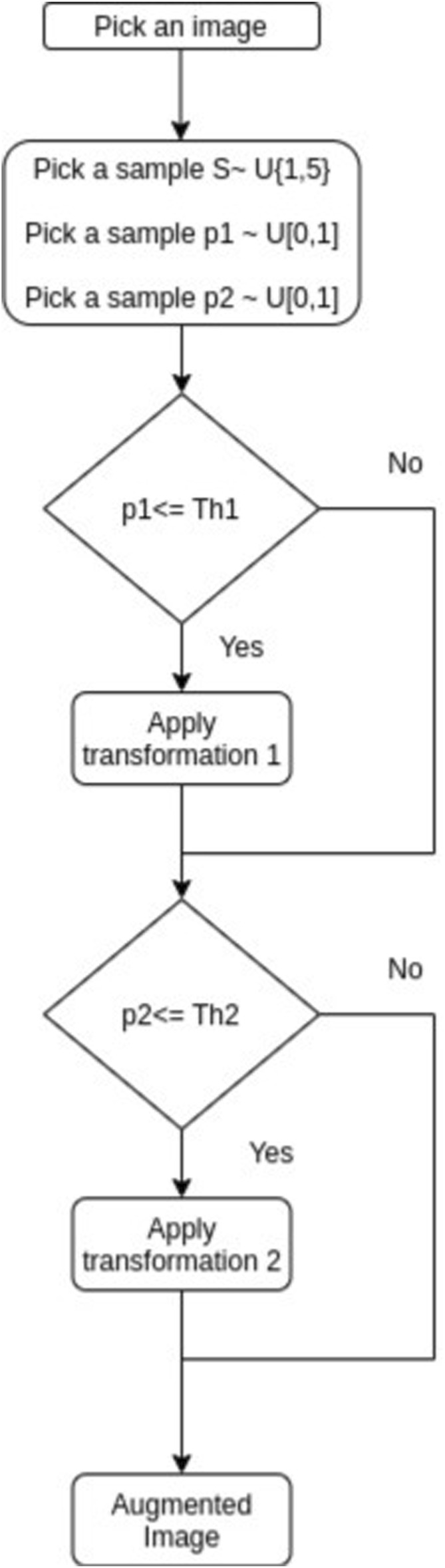
Data augmentation strategy from Zoph et al. (2020).

The strategy for data augmentation used in this work is proposed by Zoph et al. (2020); they used reinforcement learning to find which transformations are the ones that improve the most in the performance of object detection. Figure 6 is a diagram that explains this strategy and Table 1 provides the type of operations applied to the images, where.

1) X Translation: Translates the images along the horizontal axis. The number of pixels translated was chosen uniformly at random in this application, from −60 to 60 pixels.
2) Equalization: Perform an equalization on the histogram of the image.
3) Bbox Y translation: Translate only the bounding box section along the vertical axis, from −75 to 75 pixels.
4) Cutout: Fill a square section of the image with L = 48 pixels with
     (128,128,128) pixels of grey level, and then place it randomly in the image.
5) Y Shear: Apply shear mapping along the vertical axis; the range could be from −30 to 30 pixels
6) Rotation: Rotates the image; the number of degrees of rotation ranges from −30 to 30.
7) Saturation: Multiply the saturation in the HSV colour space by a factor between .54–1.54.

**TABLE 1:** Image transformations used in the data augmentation process.

**S**	Operation 1	Th 1	Range	Operation 2	Th 2	Range
0	X Translation	0.6	(−60,60)	Equalization	0.8	—
1	Bbox Y Translation	0.2	(−75,75)	Cutout	0.8	48
2	Y Shear	1	(−25,25)	BBox Y Translation	0.6	(−75,75)
4	Rotation	0.6	(−30,30)	Saturation	1	(0.54,1.54)
5	No Operation	—	—	No Operation	—	0

**FIGURE 7:**
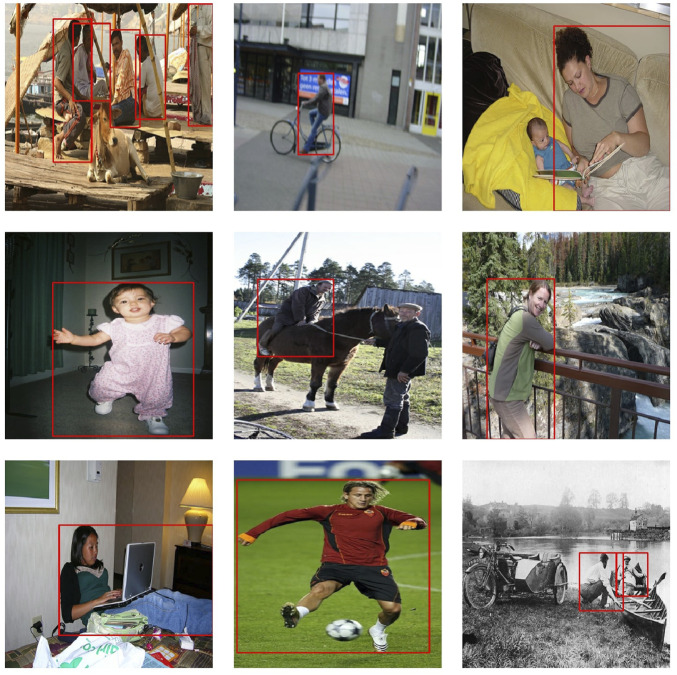
Different images from the COCO dataset and their annotations without any data augmentation technique.

**FIGURE 8:**
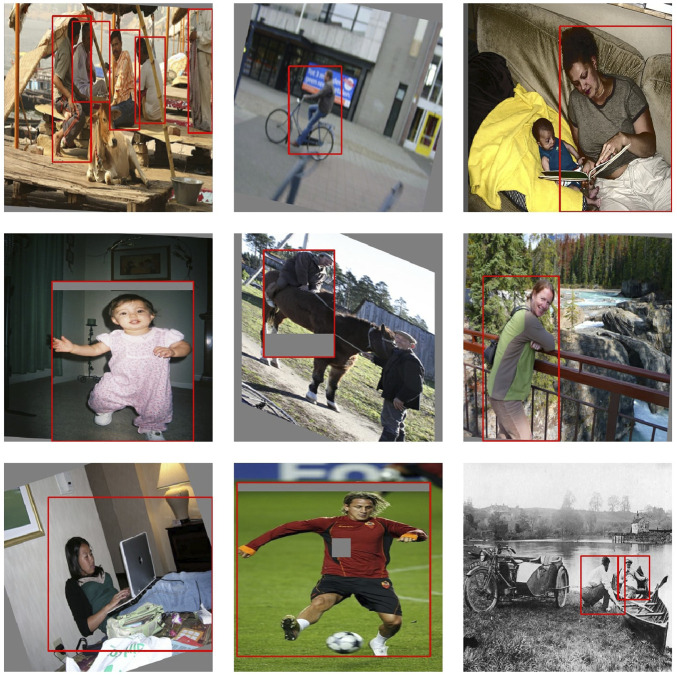
Different images from the COCO dataset and their annotations with data augmentation techniques.

Figure 7 displays different images of the training set with the annotations provided for the dataset; while Figure 8 shows the examples of the applied transformations.

## 4 Hardware selection: Devices for embedded AI applications

Deep learning models usually require enormous computational resources for training and implementation. Most of the model inference process has to be run on specialized hardware or by using clusters allocated on cloud services with powerful GPUs or TPUs. The best results achieve inference but at low frame rates (a few frames per second). However, many edge, IoT and mobile applications require processing large amounts of data without sacrificing the accuracy of model inference. Therefore, high performance inference and low power consumption are critical.

The availability of novel low-power and specialized equipment, combined with lighter but sufficiently accurate models, makes it possible to implement them in small devices. Many technical specifications are taken into account when selecting a suitable board for development. The most important technical specifications are the specific type of hardware to process and accelerate AI (GPUs, TPUs, or both): the memory available on the board (RAM and storage), the number of operations per cycle for integer (measured in GOPS, the number of Giga Operations per Second using 8-bit integers) and floating-point representation data, measured with an order of magnitud 10^9^ Floating-point Operations per Second (abbreviated as Giga-FLOPS or GFLOPS), the available embedded cameras and other sensors depending on the application, compatibility with frameworks, and power consumption levels. Table 2 shows a revision of current boards specialized for AI embedded systems. The Dev Boards presented in Table 2 are all compatible with Tensorflow or Tensorflow Lite and specialized for DNN inference in real-time, according to the information provided by the manufacturers. On the one hand, the price of different boards varies from 100 to 699 USD in the time of the searching. However, the trade-off between price and number of possible operations per cycle is best for NVIDIA Jetson Nano 4.8 GFLOPS/USD, followed by the NVIDIA Jetson TX2 with 3.3 GFLOPS/USD. For the Bitmain Sophon Edge and the Google Coral Dev Board, the ratio is 15.5 GOPS/USD and 26.8 GOPS/USD, respectively. On the other hand, the power consumption is lower for the NVIDIA Jetson Nano (5–10 W), followed by NVIDIA Jetson TX2 (7.5 W), Google Coral (10–15 W), Bitmain Sophon (24 W), and then NVIDIA Jetson AGX Xavier (30 W limit).

**TABLE 2:** Technical specifications comparison of four popular embedded systems for artificial Intelligence applications. Information obtained from the manufacturers official website.

Dev board	GPU-cores and TPU-cores	CPU	RAM (GB)	Storage (GB)	AI performance	Camera support
NVIDIA Jetson AGXXavier	512/64	Eight-core ARM v8.2	32	32 GB eMMC 5.1	32 TOPS	Yes up to 6 CSI
NVIDIA Jetson TX2	256	Quad-core ARM A57	8	32 GB eMMC 5.1	1.33 TFLOPs	Yes up to 6 CSI
NVIDIA Jetson Nano	128	Quad-core ARM A57	4	16 GB eMMC 5.1 Flash	472 GFLOPs	Yes up to 4 CSI
Bitmain Sophon Edge	Edge TPU	Quad-core A53	1	8	2 TOPS (int8)	No, supports USB Camera
Google Coral Dev Board	GC7000 Lite Google Edge TPU coprocessor	Quad-core A53	1	8 eMMC	4 TOPS (int8)	No, supports HDMI Camera

To maintain a reduced cost ratio of the platform, Jetson Nano has 4 GB of main memory which is not enough to run AI-intensive applications. Many times, when working with Jetson Nano, the screen freezes for a moment. Despite this memory size condition, it is possible to solve this problem by using swapfile, a feature of the Linux kernel used in the Jetson Nano. Swap space is the available storage area on a hard disk. It is a part of the Jetson Nano’s memory. The swap contains memory pages that are temporarily inactive. Swap space is used when the operating system decides that it needs physical memory for active processes and the amount of available (unused) physical memory is insufficient. When this happens, the inactive pages of physical memory are moved to the swap space, freeing up that physical memory for other uses, improving the response time of the Jetson Nano.

According to the information compiled, the NVIDIA Jetson AGX Xavier has the best performance, almost 20 times better than the Jetson TX2; however, the price ratio of Jetson is better for the Nano model. The NVIDIA Jetson Nano design is aimed at low-power implementations, is the cheapest of the five boards, supports many frameworks, and is intended for computer vision applications. Therefore, in this work the experimental section included the NVIDIA Jetson Nano as the hardware architecture to integrate the inference model.

## 5 Experimental results

The Transfer Learning process to fit a proposed model to a new scenario involves changing some initial and final layers, as well as adjusting parameters and hyperparameters in a training task. The training task employs Tensorflow Abadi et al. (2016), an end-to-end open-source machine learning platform, is employed in the training task. TensorRT NVIDIA (2020) improves high-performance inference on NVIDIA GPUs directly with models trained with Tensorflow. To increase performance, TensorRT converts the model to a half-precision 16-bit floating-point format; this reduces the size of the model and thus the processing time.

### 5.1 Pretrained model as feature extractor

The first experiments use the pre-trained model as a feature extractor, freezing all the model layers except the final one. We trained the model using four anchor boxes (Experiment 1) and six anchor boxes (Experiment 2) and without any data augmentation technique. Table 3 shows the results that were obtained in experiments 1 and 2. The metrics shown are the Precision (P), Recall (R), F1 Score, Objects Detected Correctly (ODC) and the Average Precision (AP) calculated for the COCO Validation set filtered with the “Person” class.

**TABLE 3:** Results obtained with step 3 experiments.

**No.**	Number anchor boxes	**P**	**R**	**F1 score**	ODC	**AP**
1	4	0.7060	**0.1854**	**0.2938**	**2041**	**0.1486**
2	6	**0.7282**	0.1687	0.2740	1857	0.1386
Original	6	0.5330	0.1584	0.2443	3273	0.1080

Results obtained with the experiments of step 3 (the best results obtained are shown in bold).

### 5.2 Fine tuning process

The following experiments used the model obtained in the previous step and used the weights as initial parameters for the training process. We experimented using four and six anchor boxes. Table 4 shown the results obtained with the percentage improvement.

**TABLE 4:** Improvement of the best models with respect to the original model.

**No.**	Number anchor boxes	**P**	**R**	**F1 score**	ODC	**AP**
3	4	0.612	0.2815	0.3856	3098	0.2026
		**+14%**	**+71%**	**+57%**	**+77%**	**+87%**
8	6	0.6744	0.2628	0.3782	2892	0.2149
		**+25%**	**+68%**	**+55%**	**+65%**	**+99%**
Original	6	0.533	0.1584	0.2443	1744	0.108

Improvement of the best models with respect to the original model (the best results obtained are shown in bold).

**FIGURE 9:**
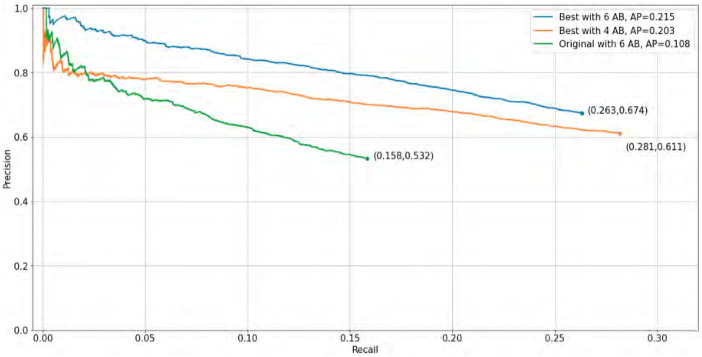
Average precision curve of our best results vs. original implementation.

Using the results obtained previously and employing four and six anchor boxes, we plot the precision vs. recall curve (Figure 9).

### 5.3 Detection results

**FIGURE 10:**
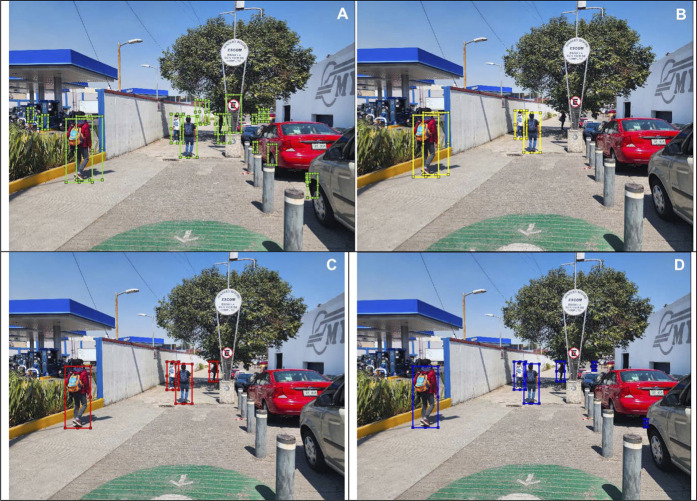
**(A)** Original image with ground truth annotations. **(B)** Detected bounding boxes by the original model. **(C)** Detected bounding boxes by the four anchor box refined model. **(D)** Detected bounding boxes by the six anchor box refined model.

**FIGURE 11:**
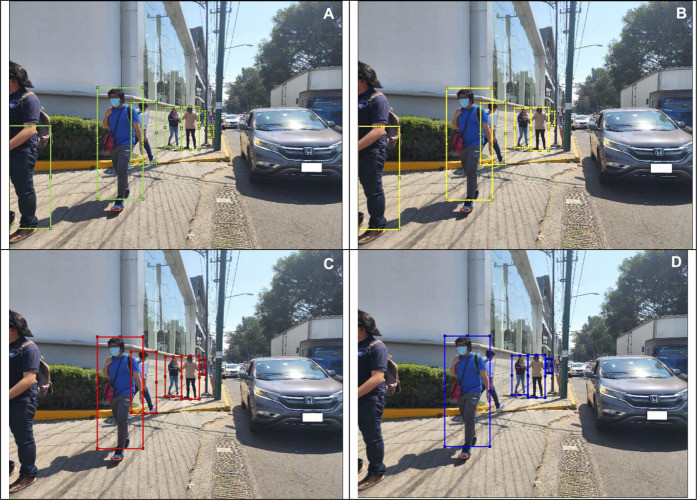
**(A)** Original image with ground truth annotations. **(B)** Detected bounding boxes by the original model. **(C)** Detected bounding boxes by the four anchor box refined model. **(D)** Detected bounding boxes by the six anchor box refined model.

**FIGURE 12:**
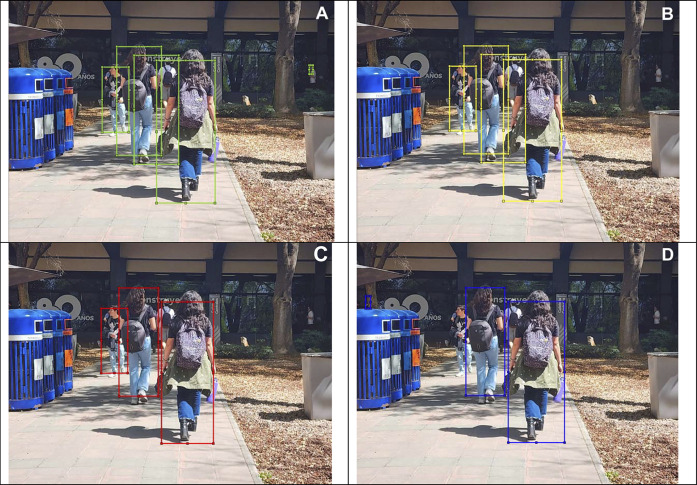
**(A)** Original image with ground truth annotations. **(B)** Detected bounding boxes by the original model. **(C)** Detected bounding boxes by the four anchor box refined model. **(D)** Detected bounding boxes by the six anchor box refined model.

**FIGURE 13:**
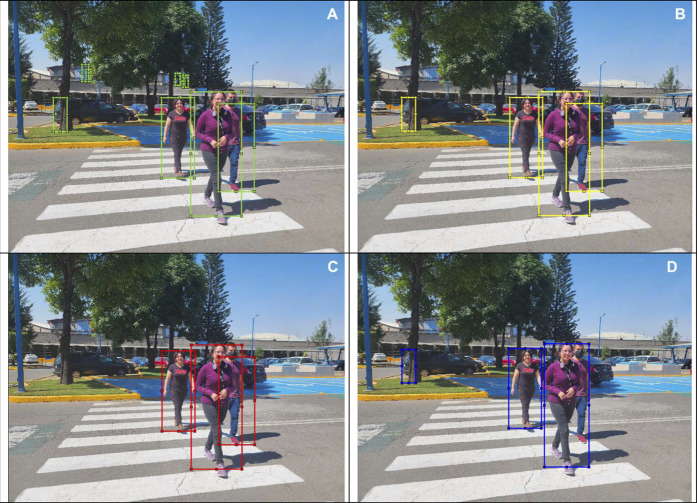
**(A)** Original image with ground truth annotations. **(B)** Detected bounding boxes by the original model. **(C)** Detected bounding boxes by the four anchor box refined model. **(D)** Detected bounding boxes by the six anchor box refined model.

In this section, we show different images that were tagged manually an placed on Figures 10–13. Each figure shows the original annotated image, the image with the original predictions of the network, the image with the predictions of the best result of the four anchor boxes, and the image with the predictions of the best result of the six anchor boxes.

### 5.4 Inference process

For the training and testing process, we used a desktop computer with the following hardware specifications.

1) CPU: Intel i7-9700k 8 cores at 3.6 GHz
2) GPU: NVIDIA RTX 2070 SUPER
3) RAM (GB): 32 GB at 2666 MHz.
4) OS: Ubuntu Linux 18.04

The following results are obtained using 416 × 616 × 3 dimension images and the reported time is in ms. Using the frozen graph, we test our refined model and the original implementation on different systems. Table 5 shows the results obtained using the CPU from the desktop computer and the CPU from the Jetson Nano.

**TABLE 5:** Performance results in CPU.

	**i7 9700k**		**ARM Cortex M53**	
	Inference time (ms)	Images/sec	Inference time (ms)	Images/sec
Refined	21	47	470	2.1
Original	750	1.3	3000	0.33

We can observe an improvement in the inference time compared with the original implementation, both on the desktop CPU and on an embedded CPU.

Using the TensorRT library, we optimized the model for a Desktop GPU and for the Jetson nano GPU. Table 6 shows the results obtained.

**TABLE 6:** Performance results in GPU.

	**Tegra X1**		**RTX 2070S**	
	Inference time (ms)	Images/sec	Inference time (ms)	Images/sec
Refined	110	9	2.8	360
Original	1200	0.8	4	250

### 5.5 Power consumption measurement

We used Jetson Nano as a case study of a low power consumption embedded system. Measuring consumption is important because the application will be used in mobile computing for visually impaired users, where managing the energy consumption values used will allow adjustments to be made to the device’s operating time.

To perform the power consumption evaluation for object detection in a video, the Jetson Nano performs the entire preprocessing task: it reads the video frame, resizes it to the image size used for the neural network, performs the neural network operations, makes the bounding box predictions, draws the rectangles in the video frame, and finally sends it to a web browser for display.

**FIGURE 14:**
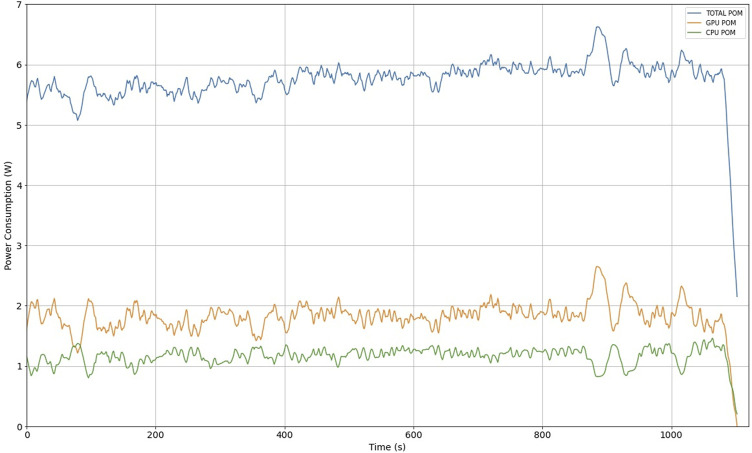
Jetson Nano power consumption measurement.

Power consumption monitoring (POM) was performed when Jetson Nano was processing the object detection task in a video. The measurement is performed using the tegrastats command-line utility which reports CPU, GPU and memory usage for Tegra-based devices like the Jetson Nano Bonghi (2020); Figure 14 shows the total power consumption of the Jetson Nano (blue) and the individual consumption of the CPU (green) and GPU (orange).

## 6 Discussion

Looking for the best performance in inference run-time on GPU devices, the selected network is suited to execute the pedestrian detection routine. Table 6 shows the performance of the original model and the tuned model for pedestrian detection; the original implementation performed worse on a CPU. Also, for devices with GPUs (e.g., the Jetson series or data centers where it is possible to rent a GPU cluster), the tuned model may give the best performance. However, if we want a model for a similar purpose (pedestrian detection) but for mobile or embedded devices where no GPU is available (e.g. Raspberry Pi, digital signal processors or high-performance microcontrollers), a different base model must be selected.

In order to make a comparison of solutions related to the implementation of assistive devices for visually impaired people, a search for articles with the perspective addressed by this work was carried out. After the use of author’s keywords, paper selection strategy use the following exclusion criteria elements: 1) Papers are not relevant to the primary research goal (by reading of abstract, introduction and conclusion). 2) Papers published in personal blogs, patents or non-academic web pages. 3) Documents without solution for obstacle detection and/or object recognition, 4) Solutions that are not implemented, and 5) Not written in English. Table 7 provides the output of such comparison, where pedestrian detection is touched from different perspectives, considering the computer vision technologies to analyze and understand a scene as the main element of discernment. Correlation column indicates the weakness or strong relationship of the research reported on those manuscripts placed on Table 7 with the one provide here, based on the declared contributions and author keywords. In this way, the focus was determined to seek solutions to meet the visually impaired requirements.

**TABLE 7:** Comparison of related reported implementations, with the following keywords abbreviations: pedestrian detection (PD), visually impaired (VI), Tiny YOLOv3 (TY), deep learning (DL), wearable assistive devices (WAD), graphic processing unit (GPU), power management (PM), image processing (IP).

**References**	Title of related work	References keywords	Strong relationship	Weak relationship
Xu et al. (2022)	An Efficient Pedestrian Detection for Realtime Surveillance Systems based on Modified YOLOv3	YOLOv3, CNN, K-means - Pedestrian detection, Shuffle unit video surveillance	TY, DL	VI, WAD, PM
Khan et al. (2020)	An AI-Based Visual Aid With Integrated Reading Assistant for the Completely Blind	Blind people, completely blind, electronic navigation aid, Raspberry Pi, visual aid, visually impaired people, wearable system	VI, WAD, IM	TY, DL, GPU, PW
Tapu et al. (2017)	A computer vision-based perception system for visually impaired	Obstacle detection, BoVW/VLAD image - representation, relevant interest points, A-HOG descriptor, visually impaired people	VI, WAD,IP, PD	TY, DL, GPU, PM
Shen et al. (2022)	A Wearable Assistive Device for Blind Pedestrians Using Real-Time Object Detection and Tactile Presentation	Wearable devices, object detection, SMA, tactile display, model compression, assistance for visually -impaired people	TY, VI, WAD, IP	PD, GPU, PM

The work of Xu et al. (2022) focus on a model to solve real-time pedestrian detection, using YOLOv3 models and modified ShuffledNet to attend security. Correlation with this manuscript deals with the manage of YOLOv3 models only. In terms of visually impaired users assistance, Mancini et al. (2018) reports a mechatronics and haptic systems, whose operations are supported by a monocular vision-based system to assist people during walking, jogging, and running in outdoor environments. To assist visually impaired people, Khan et al. (2020) describe the use of wearables for blindness by using an embedded system, supported by Raspberry Pi 3 Model B+ and artificial intelligence. The detection of obstacles and classification methodology, using generalization support to identify objects as static and dynamic obstacles, is reported by Tapu et al. (2017). It is important to note that no information was found in the articles on how power consumption measurement intervenes in the pedestrian detection process, an important issue to consider in mobile assistive devices.

The power consumption measurement reported in Figure 14 indicates that we should consider an average power consumption of 6 W during pedestrian recognition operation. For a practical use case in mobile devices, the use of a 5000 mA h battery, at a voltage of 5 V, results in 25 W/h. For the purposes of this work, this represents an operation of a little more than 4 h using the Jetson Nano, which can be used in trajectories with different pedestrian scenarios. However, other power consumption considerations must be considered, such as the audible or mechanical guidance units included in the assistive device for the visually impaired to review autonomy.

The base model was refined and focused on the detection of one class. We observed an improvement in the number of detected objects and a decrease in the number of false positives, indicating better recall and accuracy. With the chosen metrics, we were able to measure the performance of the model, which gives us a good understanding of its strengths and weaknesses. It helps to understand the differences in performance between models with four and six anchor boxes and to choose the best model based on average accuracy. In terms of average accuracy, six anchor boxes perform better because objects with higher confidence get better definition of their box dimensions. We can observe the results in Figure 9 and the real implementation, comparing original YOLOv3 with the proposal of this work, in a crowded pedestrian street located in Mexico City https://youtu.be/ZnYK9ibM7kg.

The original and the refined models (with four and six anchor boxes) were tested using the validation set of the COCO dataset, comprising 11004 objects distributed over 2634 different images. The evaluation of the fitted model used as a feature extractor achieved a accuracy of 0.73 using six anchor boxes. However, type II errors (false negatives) measured a recall of 0.17 and an F1 score of 0.27. In contrast, fitting the model with retraining epochs results in a good balance between the 0.67 accuracy and 0.27 recall. It is important to note that accuracy penalizes type I error (false positives). Therefore, the best F1 score of the fitted model was achieved by using six anchor boxes in a retraining for the pedestrian detection class.

According to the experimental results, the model tuned only for pedestrian detection is suitable to run on different hardware configurations. For example, we get about 44% improvement in inference time using a desktop CPU that could be like those found in data centers. With an embedded device, such as a Jetson Nano, we obtained better performance than the original. In this case, nine frames per second may not be useful in an application requiring dense processing. However, using a sparse basis, the adjusted model can process enough frames to achieve real time. In addition, the fitted model is useful in embedded applications with on-board processing. That is, applications where the system does not request cloud services for processing or where information protection is crucial, and all processing must be done at the edge, which could not be possible with other embedded devices without a GPU.

A disadvantage found in the Jetson Nano was the limitation of RAM resources needed in the inference process to allocate the intermediate tensors in the network. One solution to this restriction was to change the model to work with smaller images; however, the dimension of the intermediate tensors tends to shrink, affecting detection performance.

## 7 Conclusion

This paper presents an evaluation of the Tiny YOLOv3 network for its implementation in low-cost wearable devices as an alternative for the development of assistive technologies for the visually impaired. The selected network, Tiny YOLOv3, is an architecture that is originally capable of detecting 80 different classes. For this evaluation, the model was adjusted to detect a single class (pedestrian) using Tensorflow. The parameters and hyperparameters were tuned to detect pedestrians using different anchor boxes to achieve sufficiently good performance. The tuned model achieved an improvement of 55% in the F1 Score compared to the original model. In addition, the tuned model was evaluated using the standard images from known datasets and real-world images.

The tuned model was optimized for use in the Jetson Nano embedded system, a case study for low-power embedded devices, and in a desktop computer. In the case of the embedded system, the original implementation was able to process an image on the GPU of the Jetson Nano board in 1.2 s, while the model tuned only for pedestrian detection was able to process it in about 110 m. Furthermore, using the embedded system CPU, the original implementation was able to process an image in 3 s, while the tuned model completed the same task in 470 m. Desktop tests were performed on an RTX 2070S graphics board, the original model completed the task in 0.75 s, and the tuned model completed the same task in about 21 m. These results demonstrate that the tuned model can process frames acquired by a video camera with sufficient dispersion to achieve real-time pedestrian detection.

## Data Availability

The raw data supporting the conclusion of this article will be made available by the authors, without undue reservation.
